# Analysis of red blood cells from peripheral blood smear images for anemia detection: a methodological review

**DOI:** 10.1007/s11517-022-02614-z

**Published:** 2022-07-15

**Authors:** Navya K.T., Keerthana Prasad, Brij Mohan Kumar Singh

**Affiliations:** 1 Manipal Institute of Technology, Manipal Academy of Higher Education, Manipal, India 576104; 2 Manipal School of Information Sciences, Manipal Academy of Higher Education, Manipal, India; 3 Department of Pathology, Kasturba Medical College, Manipal Academy of Higher Education, Manipal, India 576104

**Keywords:** Peripheral blood smear, Red blood cells, Image processing, Computer-aided system, Anemia diagnosis

## Abstract

Anemia is a blood disorder which is caused due to inadequate red blood cells and hemoglobin concentration. It occurs in all phases of life cycle but is more dominant in pregnant women and infants. According to the survey conducted by the World Health Organization (WHO) (McLean et al., *Public Health Nutr* 12(4):444–454, 2009), anemia affects 1.62 billion people constituting 24.8% of the population and is considered the world’s second leading cause of illness. The Peripheral Blood Smear (PBS) examination plays an important role in evaluating hematological disorders. Anemia is diagnosed using PBS. Being the most powerful analytical tool, manual analysis approach is still in use even though it is tedious, prone to errors, time-consuming and requires qualified laboratorians. It is evident that there is a need for an inexpensive, automatic and robust technique to detect RBC disorders from PBS. Automation of PBS analysis is very active field of research that motivated many research groups to develop methods using image processing. In this paper, we present a review of the methods used to analyze the characteristics of RBC from PBS images using image processing techniques. We have categorized these methods into three groups based on approaches such as RBC segmentation, RBC classification and detection of anemia, and classification of anemia. The outcome of this review has been presented as a list of observations.

**Graphical abstract:**

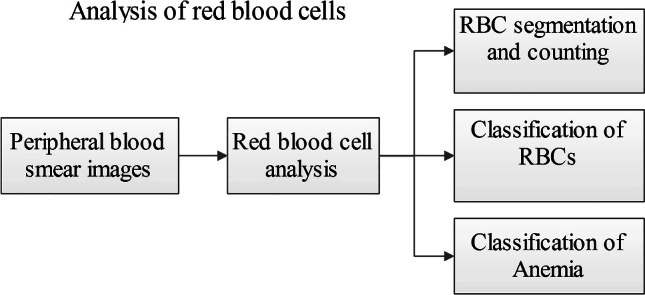

## Introduction

**Fig. 1:**
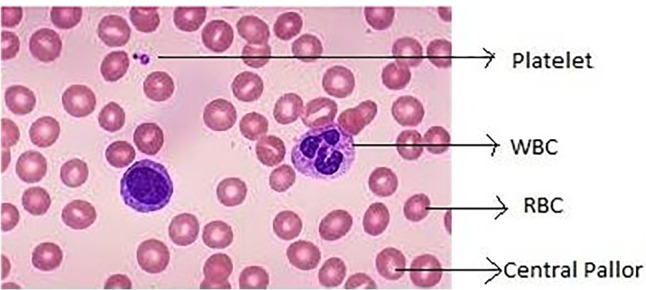
Microscopic view of blood smear image [95]

Anemia is a condition described by insufficient red blood cells or based on hemoglobin content in the blood below a specific range estimated for specific sex and age of a person. Anemia is diagnosed using PBS where microscopic examination of blood smear provides useful information about alteration of RBC shape and size or presence of any inclusion bodies. RBC morphology is a key tool for hematologists to recommend appropriate clinical and laboratory follow-up and to select the best tests for definitive diagnosis. Anemia analysis can be done based on RBC morphology and clinical parameters. Morphological analysis using blood smear is performed by spreading a drop of blood thinly onto a glass slide and stained with coloring agents such as Giemsa, Leishman, and Wright-Giemsa and examined under a microscope by a qualified lab technician [93]. The blood smear contains different types of cells, namely White Blood Cells (WBCs), RBCs and platelets. An image of PBS indicating different blood cells is shown in Fig. 1.

**Fig. 2:**
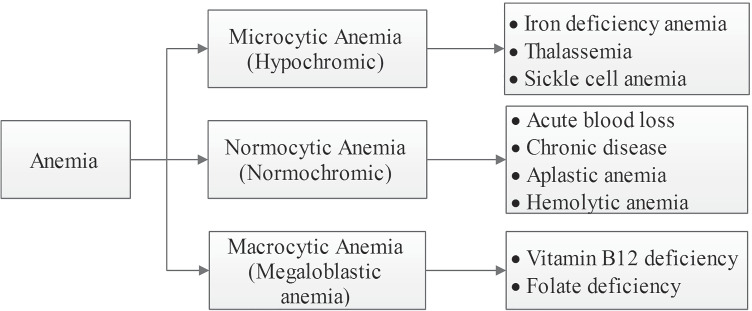
Classification of anemia

It can be observed that RBCs are more in number in comparison with WBCs and platelets. During this examination of the smear, the pathologists assess the size, shape, and color of the RBCs and WBCs. Also, they estimate the number of platelets present. The quality of RBC is characterized by red cell indices and any deviation in size, volume, or shape of red cells represents an abnormal red blood cell [78]. Anemia is classified based on the morphology of red cells, red cell indices and hemoglobin content as in Fig. 2.

**Fig. 3:**
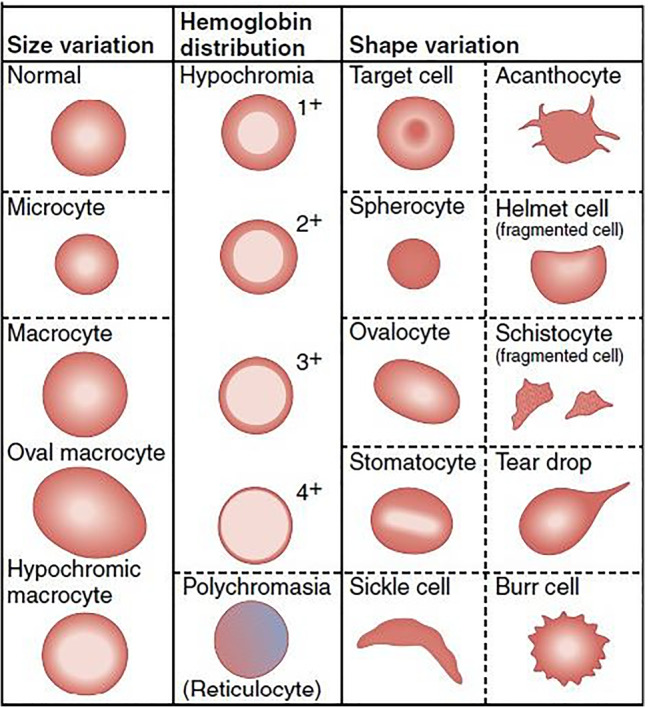
Normal and abnormal RBCs [95]

Anemia is classified into hypochromic microcytic, normochromic normocytic and macrocytic anemia. Further, it is categorized into Iron Deficiency Anemia (IDA), Sickle Cell Anemia (SCA), thalassemia, Hereditary Spherocytosis (HS), Hereditary Elliptocytosis (HE), aplastic anemia and Hemolytic Anemia (HA) based on the RBC morphology. Based on the morphology, types of normal and abnormal red blood cells are shown in Fig. 3.

Anemia classification can also be performed based on the clinical parameters such as RBC count, RBC indices, namely Mean Corpuscular or Cell Volume (MCV in femtoliter), Mean Cellular Hemoglobin Concentration (MCHC), Mean Cell Hemoglobin Content (MCH in picograms), hematocrit (HCT) or Packed Cell Volume (PCV) and Red Cell Distribution Width (RDW). These parameters play an important role in the detection and classification of anemia. Hematologists usually examine PBS if RBC indices are abnormal [58]. The morphological classification of anemia based on the clinical diagnosis is as shown in Table 1.

**Table 1:** Classification of anemia based on the clinical parameters

Anemia Type	MCV fL	MCH pg	MCHc%
Macrocytic anemia	> 100	> 32	32–35
Normocytic anemia	80–100	27–32	32–35
Microcytic anemia	< 80	< 27	< 32

This paper presents a comprehensive review of automation of PBS images for detection of anemia. Many research groups have attempted this automation based on clinical or morphological analysis.

## Approaches

This survey summarizes the various research works involved in the automation of analysis of the PBS images. The approaches are categorized as RBC segmentation and counting, RBC classification and detection of anemia, and anemia classification.

### RBC segmentation and counting

In this section, we provide information about RBC segmentation and counting using image processing methods based on color and size variations. The segmentation techniques are categorized into different sub-sections based on the approaches.

#### Thresholding and transform-based segmentation methods

Thresholding is the simplest way of segmenting an image into foreground and background based on the different intensities or colors. Transform methods are used to identify the features in the other domains. Prasad et al. [124] developed a decision support system to detect malaria parasites in thin PBS images using color image analysis. Morphological operations were used to detect RBCs and color image processing techniques to extract the region of interest. This method could detect around 96% of the parasites for 200 Giemsa stained images of 100X magnification under uniform stain and illumination conditions [58] [90, 125]. Bhavnani et al. [40] proposed a method to segment and count RBCs and WBCs using Otsu thresholding and morphological operations. WBC counting was performed by counting number of connected components and obtained an average accuracy of 94.25%. RBC counting was performed using Watershed segmentation and Circular Hough Transform (CHT) and accuracies of 92.67% and 91.07% were obtained respectively. The principal objective of watershed segmentation [87] is to find the watershed lines which forms continuous path giving rise to continuous boundaries between the regions. It extracts nearly uniform objects from the background. CHT is an image transform [53] that extracts circular objects from an image. The transform can measure radius and the centroid of each circular object in an image by searching a 3D Hough space. Maji et al. [107] presented RBC counting method using Otsu thresholding and mathematical morphology and classified into circular, non-circular, overlapped cells or artifacts. The average accuracy obtained was 96.9% for circular and 97.1% for non-circular cells from 146 images. Ruberto et al. [65] proposed a method for malarial parasite-infected blood cells using HSV component based on color similarity and Watershed algorithm for 12 Giemsa stained images acquired at different magnifications with some variations in stain and lighting conditions. Ruberto et al. [66] proposed a method based on region proposal using edge boxes for detecting and quantifying RBCs and obtained accuracy in the range of 96–98% for 180 ALL-IDB images. The research groups [67] presented the same method for another malarial parasite MP-IDB database with 100 images and achieved accuracy in the range of 89–99%. Sharif et al. [144] presented a preliminary study on RBC segmentation method using masking and watershed algorithm for 20 images with 40X magnification. However, this method needs improvement in segmentation for large overlap. Biswas et al. [43] proposed blood cell segmentation method using Watershed Transform (WT) [118, 144] and Sobel filter in the spatial frequency domain [60, 126] and obtained 93% accuracy for 30 images measured using a structure similarity index matrix. Habibzadeh et al. [86] proposed a method for WBC and RBC segmentation using YIQ color space and WT and 90% accuracy was achieved for RBC segmentation using 10 images. However, they reported addressing large variations of blood cells and low quality images in their future work. Cruz et al. [57] proposed RBC counting method using blob analysis based on HSV component and WT and obtained an average accuracy of 95.6% for 10 blood samples taken with 40X and 100X magnifications. Segmentation of RBCs from PBS images using Hough Transform (HT) was reported by many research groups [14, 72, 83, 110, 113, 149, 155]. They reported the accuracy in the range of 94–96%. Mahamood et al. [104, 105] proposed color based blood cell segmentation in CIELAB color space and used CHT for cell extraction. The experiment was performed on ALL-IDB dataset with 108 Wright stain images of magnification ranging from 300X to 500X and obtained the average accuracy of 81% for WBCs and 64% for RBCs. Sarrafzadeh et al. [139] presented a circlet transform-based method to count RBCs and obtained a low error rate for 100 images with 100X magnification. However, the authors suggested to improve initial RBC mask for accurate segmentation. Yeldhos et al. [161] implemented FPGA based embedded system for counting RBC using CHT. YCbCr color conversion and WT segmentation method were used. An accuracy of 90.98% was achieved for 108 blood smear images of ALL-IDB dataset. Frejlichowski [80] proposed a method to detect RBCs based on pixel relationship and obtained 83% accuracy for 700 RBCs from May-Grunwald-Giemsa (MGG) stained images. Alomari et al. [28] proposed an iterative structured circle method to detect WBCs and RBCs and obtained average accuracy of 95.3% for RBCs and 98.4% for WBCs from 100 images of different magnifications ranging from 300 to 500X.

#### Edge based segmentation methods

Das et al. [60] proposed a method to identify RBCs and different types of WBCs using edge detection algorithms, namely Canny, Laplacian of Gaussian (LOG), Sobel and obtained 85% accuracy for 20 images. Poomcokrak et al. [123] proposed Canny edge algorithm based RBC counting method. The method obtained 74% accuracy for 59 RBCs and 59 non-RBCs using Multilayer Perceptron (MLP). MLP is a simple feed forward neural network that uses back propagation algorithm to train neurons [89, 92]. It consists of an input layer, an arbitrary number of hidden layers and an output layer. The hidden layer processes the input information and transmits to the output layer. MLPs are often applied to supervised learning problems. Backpropagation is used to adjust weights and biases to minimize the error. Hafiz et al. [19] proposed RBC segmentation algorithm using boundary-based thresholding and Canny edge detection methods and obtained average accuracy of 87.9% for five images from the Broad Bioimage Benchmark Collection (BBBC) dataset.

#### Clustering based segmentation methods

Abbas et al. [11] presented a method to segment blood cells using the YCbCr color space and K-means clustering method [45] for 90 Giemsa stained blood smear images. Blood cells were easily identified by this method using a unique color of every component. Wei et al. [157] proposed a method to detect and count overlapped RBCs in microscopic blood smear images. The H and S components were used to differentiate between WBCs and segmented RBCs. H and S components are closely related to the way humans feel color. H is the color sensed due to the wavelength. S indicates the purity of the color [126]. Watershed and K-means clustering algorithms were applied for segmentation. An accuracy of 92.9% was obtained for 100 Wright-Giemsa stained images. However, the authors of this paper suggested to fine tune the segmentation method for robustness. Acharya et al. [15] presented a method to separate RBCs from other components of blood using K-medoids and obtained 98% accuracy for 1000 Wright stained images. Savkare et al. [140] proposed a method to segment blood cells using K-means clustering algorithm and WT and obtained 95.5% accuracy for 78 Giemsa stained microscopic images. However, they reported that if cells are not well-stained and have low contrast, this method does not work well. Ruberto et al. [68] presented a fuzzy set based optimal threshold selection approach for blood cell segmentation. The local threshold was set using a histogram and average accuracy of 98% was obtained with a computation time less than a second.

#### Contour and matching based segmentation methods

Bronkorsta et al. [46] proposed a parametric deformable template-based online detection method to detect RBC shapes of 900 cells in 100X magnification and obtained accuracy of 95.7% for 10 images. This technique is based on the prior knowledge about the shape and appearance of the object. A template prototype and according energy function is defined for template description. However, a good initial guess for the shape, size, and location of the object is needed to find global minimum in this method. Bergen et al. [39] proposed a method for WBC and RBC segmentation using template matching and level set algorithm [141]. A Dice coefficient score of 0.96 was obtained for WBC segmentation from 155 images. Ritter et al. [133] proposed a blood cell segmentation method using a graph algorithm and obtained a success rate of 90% for 98 images. However, due to the diffuse area, this method failed to detect all platelets. Cai et al. [47] presented an RBC segmentation method based on an active appearance model incorporating shape and texture information of the cell.

#### Machine learning-based segmentation methods

Sadafi et al. [135] presented a fully convolutional neural network-based RBC segmentation method and obtained 90% accuracy for 5772 raw images of different stains. In this work, the authors suggested to use post-processing methods for touching cell split up to improve the accuracy. Kimbahune et al. [98] and Jun et al. [108] proposed blood cell image segmentation and counting method using Pulse-Coupled Neural Network and found that the method is time efficient. A machine learning approach based on the YOLO algorithm was presented by Alam et al. [20] to identify and count blood cells and obtained an accuracy of 96.09% for RBCs and 86.89% for WBCs from 364 100x magnified annotated images of Blood Cell Count Dataset (BCCD). Adagale et al. [16] proposed an overlapped RBC counting algorithm using Pulse Coupled Neural Network with a template matching technique and obtained 90% average accuracy for 40 images. Chari et al. [51] presented a pilot study on the analysis of MGG stained normal images using Shonit^*TM*^ artificial intelligence system. The extracted cells were classified using three different deep neural network models based on images annotated by three experts. The precision of 93.9% was achieved for all WBC classes from 6000 WBCs. RBCs and platelets were identified based on the estimation of indices for 100 images from every 100 samples and obtained estimation within 10% reported value of Sysmex XN 3000^*TM*^ hematology analyzer. Loddo et al. [101] proposed a blood cell counting method using nearest neighbor and SVM techniques. This method used ALL-IDB dataset with 368 images and obtained an average accuracy of 99.2% for WBCs and 98% for RBCs. Tran et al. [150, 151] presented deep learning semantic segmentation method for RBC and WBC segmentation and counting. It is pixel level segmentation of the image. An experiment was conducted on 42 ALL-IDB database images with 380 training images post augmentation. SegNet architecture was utilized to segment blood cells by labeling each pixel. It is a deep architecture for the segmentation of multi-class based on assigning each pixel of an image into a corresponding class [38]. The segmentation accuracy for WBCs, RBCs and the background reached 94.93%, 91.11% and 87.32%, respectively. For cell counting, Euclidean distance transform and binary dilation were used and accuracy of 93.3% for RBCs and 97.29% for WBCs was obtained. Shahzad et al. [143] designed semantic segmentation using a convolutional encoder-decoder framework along with VGG16 network and the model was trained and tested on the ALL-IDB dataset with 108 images. The proposed system achieved accuracies of 97.45%, 93.34%, and 85.11% for RBCs, WBCs, and platelets respectively. Amin et al. [33] presented a comparison of different classification algorithms using WEKA tool for hematological data. The experiment was conducted on two datasets from a total of 900 samples with CBC parameters. Three data mining classifiers were tried, namely J48 decision tree, MLP, and Naive Bayes, using which accuracies of 97.2%, 86.6% and 70% were achieved respectively.

#### Miscellaneous category

Gupta et al. [84] identified RBCs using blob detection method and obtained 75% accuracy for 88 RBCs. However, a few RBCs were left undetected due to the lighting conditions. Hidalgo et al. [81] proposed a novel method to count the number of circular and elongated RBCs using circumference and ellipse adjustment algorithms for 66 Giemsa stained images from erythrocytesIDB database. They used k-curvature for separating clustered RBCs and obtained 98% accuracy without pre-processing steps. Hegde et al. [90] presented a review on WBC, RBC, platelet analysis techniques and highlighted the importance of illumination and color shade variation correction to develop a robust system for PBS analysis.

**Fig. 4:**
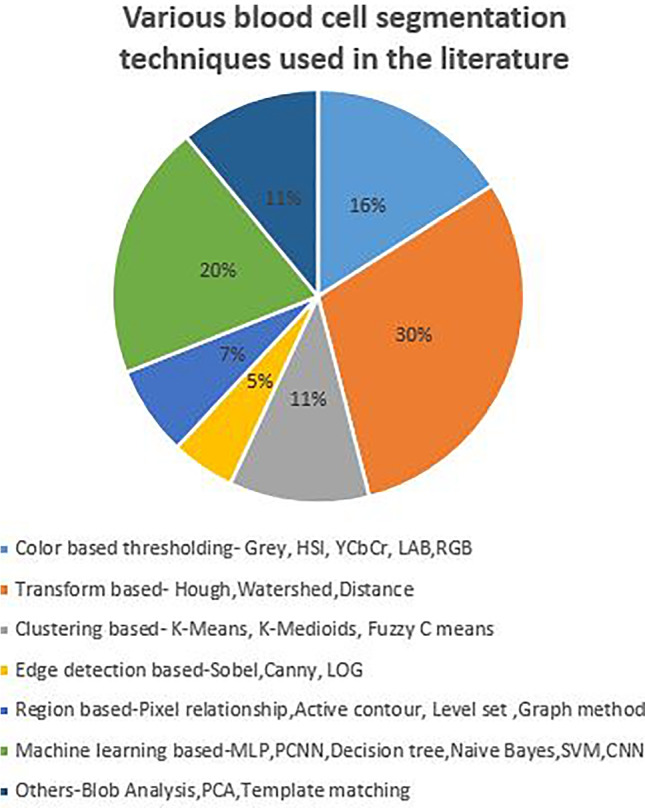
Distribution of blood cell segmentation methods

A lot of work has been carried out to segment blood cells from PBS images. The distribution of segmentation techniques used in the literature is depicted in Fig. 4.

It is observed from the distribution that most of the literature used transform method, color thresholding and machine learning techniques for the segmentation. The summary of the literature described in Section 2.1 highlighting results of each method which is listed in Table 2.

**Table 2:** RBC segmentation and counting methods

Methods	No. of images(Stain)	Accuracy(%)	Remarks	Ref.
K-means clustering, WT	60 (Giemsa )100 (Wright–Giemsa)	93–98.9	Robustness is not explained	[11, 140, 157]
Iterative structured circle detection, circlet transform	100	95.3	Incorrect hole filling leads to errors To improve initial RBCs mask for accurate segmentation	[28, 143]
Graph algorithm	98	99	Considered only non-overlapped cells	[133]
Parametric template matching, PCNN	900 cells	90–95.7	Require prior knowledge about the appearance of the cell	[16, 46, 98]
YOLO algorithm	364	96.1	Satisfactory performance	[20]
HSV conversion, morphological operations	200 (Giemsa)	96	Used uniform staining and illumination	[125]
Pixel relationship	10 (MGG)	83	Occluded objects are rejected before the later stages	[80]
Canny, LOG, Sobel	20–30	85–93	Normal RBCs Less samples	[43, 60]
K-curvature, circumference and ellipse adjustments	66	98	Images are not preprocessed to reduce execution time	[81]
Blob analysis, WT	10 blood samples	90–96	Need optimization to get accurate results	[57, 86]
CNN AlexNet	5772	90	Average execution time was 227 ms	[135]
Canny edge, MLP	59 RBCs and 59 non RBCs	74–88	Increase training images	[123] [19]
K-medoids, distance transform	1000 (Wright)	98	Processing of central pallor of RBCs consume more time	[15]
HT	500 subjects	91–94.9	Many tunable parameters	[72, 110, 148, 155]
Deep neural network models	100 (MGG)	Indices lie within the 10% of Sysmex reported value	Considered only normal blood smear images	[51]
CHT, NN, SVM	368	98	Achieved low false negative rate	[101]
LAB, YCbCr color space, CHT	108(Wright)	81–91	Computational time is more	[105] [161]
Region proposal	180 (Wright)	96-98	Tested on ALL-IDB and MP-IDB datasets	[65]
Semantic segmentation	108 (Wright)	91–97	More labeled images are required	[143, 150, 151]

### RBC classification and detection of anemia

In this section, we provide the details of image processing techniques used to classify RBCs based on shape, size and texture variations in order to detect anemia.

Classification of RBCs into normal and abnormal was presented using image processing techniques [18, 23, 24, 54, 69, 99, 112, 121, 127, 147, 148, 154]. Various methods such as Otsu thresholding, CHT, statistical and moment invariants, and geometric texture features were used. The accuracies in the range of 83–94% were obtained using ANN, SVM and BPNN classifiers.

#### Shape feature and region based RBC classification methods

Wheeless et al. [158] presented a method to classify RBCs into normal, sickle or other abnormal cell using recursive partitioning and form factor. The recursive partitioning technique is based on the concept of finding the cutpoints for the features that best isolate different disease cases. The data are then divided according to these cutpoints [44]. Form factor provides a measure of circularity as given in equation. If more is the departure from the perfect circle value 1, lower is the form factor value.

1 $$Form Factor=(4{\pi}Area/Perimeter)^{2}$$

An accuracy of 85% for normal RBCs, 83% for abnormal cells, and 81% for sickle cells from 3878 cell images was obtained. Safca et al. [136] proposed a method to classify RBCs into sickle cells, echinocytes and elliptocytes using morphological operations and shape features such as diameter, area and perimeter [13]. Morphological imaging operations done on a binary image to remove small objects, fill the cell holes and clearing the border to avoid edge touching cells [147]. An average accuracy of 96% was achieved for 34 images. Deb et al. [64] proposed an algorithm to classify RBCs using aspect ratio and Fourier descriptors and obtained average accuracy of 92% for 33 images. The authors also presented a method to count NRBCs and WBCs based on the roundness factor. Rezatofighi et al. [132] proposed RBC detection method using polar transformation and run-length matrix and a true positive rate of 97.73% was obtained for 22 blood smears. However, objects with large size variations failed to be detected by this method. Soltanzadeh et al. [146] presented a method to classify three types of RBCs using morphological methods. The method obtained 98.63% for elliptocyte, 96.7% for discocyte and 95.36% accuracy for echinocyte recognition for 200 images based on Euclidean distance. Frejlichowski [79] proposed RBC classification method using template matching and Fourier transform. A recognition rate of 93% was obtained for 55 MGG stained images. Arnau et al. [82] presented a method for RBC classification using an active contour segmentation. This method classified RBCs into normal, sickle cells and other cell deformations and obtained 95% accuracy for 45 images. Aruna et al. [34] proposed a method to detect sickle cells using Canny Edge, LOG, Prewitt, Robert and Sobel operators and found that the Canny edge detection method was preferable. Rakshit et al. [128] presented sickle cell detection method using Sobel edge detector and region properties and obtained an overall accuracy of 95.8%. Ahmadzadeh et al. [17] presented a method to group RBCs into three clusters (biconcave, stomatocyte, and sphero-echinocyte) using K-medoids, and K-means clustering and obtained 95% accuracy for Digital Holographic Microscopic (DHM) images. Chandrasiri et al. [48, 49] presented an algorithm to identify four common types of anemia, namely elliptocytes, microcytes, macrocytes and spherocytes, using HT and morphological operations. The researchers could obtain an accuracy in the range of 91–97% based on cell features for 40 images.

#### Machine learning-based RBC classification methods

Bhowmick et al. [41] presented classification of RBCs in scanning electron microscopic images using a Marker-controlled watershed segmentation method. It is combinational approach of edge-based segmentation and morphological operation methods that uses markers on some set of norms. A marker is a connected component that can easily segment boundaries from an image. With this approach, the regional minimal values occur only at marked locations [117]. The authors projected both structural and textural feature classification in this work and obtained an accuracy of 88.99% for 132 anemic blood samples using Bayesian classifier. Bayesian approach classifies the new instance by assigning the most possible target value, given the attribute values that represent the instance on the principle of Bayes’ Theorem [122]. Das et al. [61] proposed a method for RBC characterization in anemia using Marker-controlled watershed segmentation and morphological features. The algorithm classified five different types of RBCs such as elliptocyte, echinocyte, acanthocyte, sickle cell and teardrop cell in anemia and obtained an accuracy of 86.87% for 715 abnormal and 290 normal RBCs using logistic regression classifier. A method to recognize abnormal RBC shapes such as teardrop, echinocyte and elliptocyte using Hu’s moments for 300 anemia and 100 Leishman stained images of thalassemia cases was proposed by [62]. Elsalamony [74, 75] proposed a geometrical shape signature method to detect sickle and elliptocytosis using CHT and watershed segmentation and 100% accuracy was achieved for 30 images. Elsalamony [76] proposed benign and distorted cell detection methods using HT and WT and obtained 96.9% accuracy using NN and 92.9% using Classification and Regression (C&R) tree for 180 cells from 45 images and reported that NN was preferred over C&R tree to detect sickle cells. In another paper, Elsalamony [77] used Self-Organising Map (SOM) along with the above mentioned methods and reported that SOM does not require any target variables but gets slower in training the neurons. A neural network-based algorithm was proposed by Kim et al. [97] to distinguish abnormalities in RBCs and WBCs using Principal Component Analysis (PCA) and obtained 91% average recognition rate for RBCs in classifying 12 classes from 680 RBCs. Lee et al. [100] proposed RBC classification method using a hybrid neural network and identified sickle, horn and elliptocyte cells. An accuracy of 91% was achieved by this method for a dataset consisting of 200 normal and 200 abnormal single cells. Rodrigues et al. [134] proposed a method to classify RBCs into normal, sickle cells, and erythrocytes with other deformations using morphological properties and obtained 94.6% accuracy using SVM classifier for 626 images from the erythrocytesIDB dataset. They used ANOVA for feature selection and suggested to study unsupervised methods to identify the patterns in cells. Hirimutugoda et al. [91] presented a method to detect malarial parasites and thalassemia using 3-layered ANN for 200 Giemsa images of each case and obtained 86.54% correct recognition rate by defining ROIs. Aliyu et al. [26] presented RBC classification method using SVM and obtained 100% accuracy for normal, acanthocyte, teardrop cells and 73% for elliptocyte and 90% for sickle cells using SVM and 33% using deep learning for 250 images. They reported that the SVM classifier outperformed DL due to limited datasets. A research group [24] also proposed a method to detect sickle cell using Otsu thresholding and shape features and obtained 88% accuracy for 30 Giemsa stained images. Dalvi et al. [59] proposed a method to classify RBCs into four abnormal types, namely elliptocyte, echinocyte, teardrop and macrocyte, using thirteen geometric features and achieved better accuracy using ANN than the decision tree. The accuracy obtained was 96.04% for RBC counting and 90.54% for RBC classification. Razzak et al. [130] presented contour aware segmentation method based on CNN and extreme learning. The experiment was conducted on 64,000 blood cells from ALL-IDB database. RBCs and WBCs were segregated based on the color intensity features and were cropped to extract features using CNN and given for ELM for subtypes classification. The segmentation accuracy of 98.12% and 98.16% and classification accuracy of 94.71% and 98.68% was achieved for RBCs and WBCs respectively. Mundhra et al. [116] proposed deep learning-based Shonit^*TM*^ system to localize and classify blood cells. U-net deep learning architecture was used to localize WBCs and platelets from 300 MGG and Leishman stained training images. Otsu thresholding in the green channel was used to identify RBCs and clumped cells were rejected. CNN architecture was used to classify WBCs and RBCs based on size and shape. The sensitivity for WBC extraction was 99.5%. The sensitivity and specificity of identification for the common cell types were above 91% and 98% respectively. Alom et al. [27] presented deep learning-based inception recurrent residual convolutional neural network for WBC and RBC classification. The recognition accuracies of 100% for 352 WBC images and 99.94% for 3737 RBC images were achieved. It is mentioned that the model requires a large number of network parameters. Durant et al. [71] proposed a method for RBC classification based on morphology using CNN for 10 classes. Around 3737, 100X magnified labeled cells were used and correct classification frequency of 90.60% was achieved. The researchers reported that the distribution of labels for cell types was not homogeneous.

#### Clinical parameters based RBC classification methods

Zahir et al. [162] presented an ANN-based method to detect RBC disorders anemia and polycythemia using Hb value, MCH and RBC count and obtained significant results for more than 90% of the 1000 blood samples with training time less than 15 minutes. Bacus et al. [36, 37] presented RBC classification method by extracting the features and obtained correlation coefficient of 0.965 for 100 cells from 4 specimens. Red cell indices along with the red cell differential counts were considered in this work. Maity et al. [106] presented a method to generate an anemia diagnosis report based on the CBC report and RBC morphology using red cell indices and shape features. A precision of 98.2% was achieved in classifying microcytic, macrocytic, sickle, teardrop, elliptocyte, and normal cells from 1500 Leishman blood smear images.

**Fig. 5:**
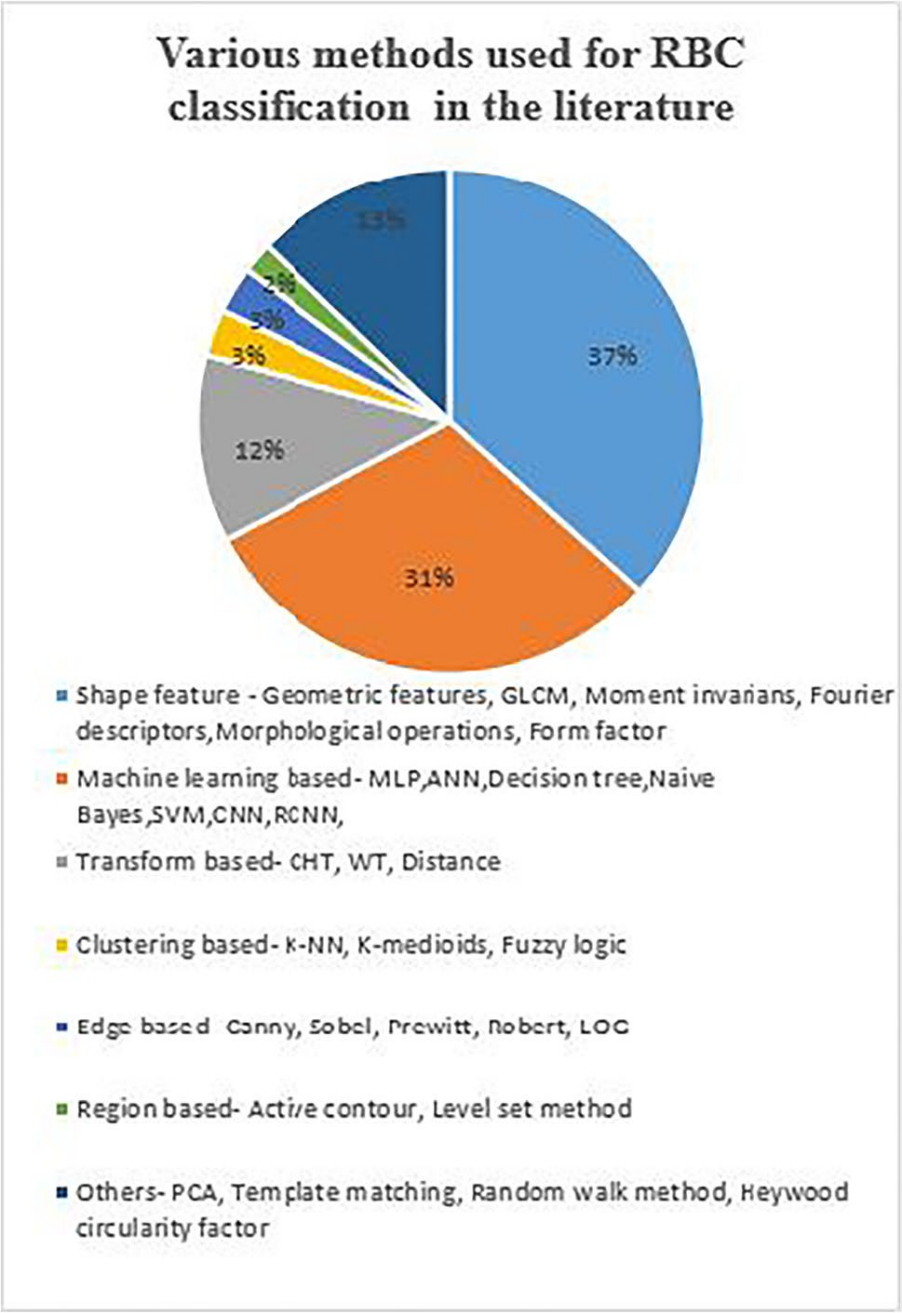
Distribution of RBC classification methods

Figure 5 shows the distribution of RBC classification methods used in the related works to detect anemia. We observe from the distribution that, majority of the researchers used shape features and machine learning to classify RBCs. A brief overview of the RBC classification and anemia detection methods are listed in Table 3.

**Table 3:** RBC classification and anemia detection methods

Methods	No. of images (Stain)	Performance metric	Remarks	Ref.
CHT, Heywood circularity factor, ANN, moment invariants, inclusion-tree structure, BPNN, PCA, SVM	150–1000 samples	80–99% accuracy for normal & abnormal RBCs	Lacks robustness	[18, 69, 147, 149, 154]
Morphological properties, Naive Bayes, K-NN, SVM, Sobel edge	626	94.6–96% accuracy for normal and sickle cells	Consider unsupervised classifiers for more RBC patterns	[128, 134]
CHT, WT, NN, decision tree, SOM, SVM	30–45 (Giemsa)	97–100% accuracy for sickle and elliptocytosis	Geometrical shape signature is used for detection process	[74–76]
Recursive partitioning, form factor	3878 cells	85% for discocytes, 83% for abnormal cells and 81% for sickle cells	Form factor invariant to cell size and provides useful information on cell shape	[121, 158]
Hybrid neural network	200 normal and 200 abnormal cells	91% accuracy for sickle, horn and elliptocytes	Considered only convexity index feature	[100]
DL, SVM	105 normal and 250 abnormal	Normal—100%, achantocyte—100%, sickle cell—90%, teardrop—100% and elliptocyte—73% accuracy using SVM	SVM classifier outperformed DL	[23, 26]
Rolling ball background, shape features, Naive Bayes, Bayesian classifier	1500 (Leishman)	98.2% precision for microcytic, macrocytic, sickle, teardrop, elliptocyte	Decision from CBC test measures is semi-automatic operation	[106]
ANN	1000 blood samples	Less computational time	Used RBG values—from Hb, MCH and RBC count	[162]
CNN , ELM	64,000 blood cells	94.71% accuracy	Images from multiple sources are used	[130]
U-Net	300 (MGG) and (Leishman)	91% sensitivity and 98% specificity	Results are shown for a variety of smear and stain	[116]
Inception recurrent residual CNN	352 WBCs and 3737 RBCs	100% for WBC and 99.94% accuracy for RBC	Model require larger number of network parameters	[27]
CNN	3737 labeled Cells	90.6% accuracy for 10 RBC classes	Label distribution was not homogeneous	[71]

### Classification of anemia

This section provides details of image processing methods used to classify anemia based on the morphology and hematological parameters of RBCs.

#### Morphology based anemia classification methods

Chen et al. [52] presented a method to classify hemolytic anemia based on differential value and variation of chain codes in eight directions and irregularity of erythrocytes. Accuracy in the range of 95–97% was achieved for 24 microscopic images using Bayes classifier, logistic model trees and rule based classifiers. Nithyaa et al. [119] proposed a method to detect various blood disorders such as malaria, elephantiasis, trypanosomiasis, SCA and polycythemia using statistical features and Euclidean distance for 40 images. Azam et al. [35] presented a method to detect seven RBC types of anemic diseases using shape descriptors and obtained 92% accuracy for 100 instances using MLP and random forest classifier. Tyas et al. [153] presented a semi-automated algorithm to classify four types of abnormal RBCs such as teardrop, acanthocytes, sickle cell and target cell using GLCM features in the minor thalassemia cases. The ROI was selected manually in this method and accuracies of 93.22% and 92.55% were obtained using BPNN and CNN respectively for 256 images. Various methods [109, 129] have been used to detect thalassemia using K-means clustering, active contour, neural network, decision tree and an average accuracy in the range 82–95% was reported. Sharma et al. [145] proposed a method to detect SCA and thalassemia using Marker-controlled watershed segmentation and geometric features and accuracy of 80.6% was obtained for 100 images with KNN classifier. Fadhel et al. [13] proposed an algorithm to count normal and abnormal RBCs in the SCA slide using CHT and WT for 233 cells and proved that CHT is better than WT. Elsalamony [73] proposed a method to diagnose SCA using HT and obtained segmentation accuracy of 99.98%. This method also achieved a classification accuracy of 96.9% and 92.9% using NN and C&R tree respectively. Lotfi et al. [102] presented a technique for the automatic detection of IDA by identifying three types of abnormal red cells using region and Fourier descriptors. This method obtained accuracies of 99%, 97% and 100% for dacrocytes, elliptocytes and schistocyte cells respectively for 100 cells of each case using NN, SVM and KNN classifier. Tyagi et al. [152] presented a method to detect poikilocyte cells in IDA using GLCM features and moment invariants and obtained accuracy in the range of 75–81% for 100 images using ANN. Several methods have been proposed to detect the sickle-shaped RBC in SCD patients by [21, 22, 29, 55, 56, 88, 114, 115, 120, 131, 159, 160] using methods such as random walk, Sobel edge, geometric features, Fuzzy C means clustering, LOG, WT, HT and morphological filters. The average accuracy reported was in the range of 85–95%. Alzubaidi et al. [30] developed deep learning models for SCA diagnosis and achieved an accuracy of 99.54%. The researchers proposed three CNN models with different layers and filters and used data from erythrocytesIDB, ALL-IDB and other internet sources. The extracted features were used for training multi-class SVM and accuracy around 98–99% was obtained. Zhang et al. [163] proposed a method to segment subtypes of RBCs in sickle cell disease using deformable U-net on 266 raw microscopy images and obtained 99.12% accuracy. This method could segment blurred, clustered and heterogeneous shaped RBCs and performed better than baseline U-net. Aliyu et al. [25] proposed the Alexnet deep learning model for the classification of RBCs in SCA. Around 750 single RBCs from Giemsa stained blood smears were acquired for the experiment and classification accuracy of 95.92% was obtained. They reported low specificity due to less normal cells. Haan et al. [85] presented a deep learning framework based screening of sickle cells using a smartphone microscope. U-net architecture for image normalization and enhancement network and semantic segmentation for sickle cells were used and approximately 98% accuracy was achieved from 96 unique patient samples. Das et al. [63] presented an overview of enhancement, segmentation and classification techniques used for SCA detection. The review also highlights clinical uses, hardware implementation and future scope for the analysis of SCD.

#### Hematological parameters based anemia classification

Birndorf et al. [42] presented ANN-based hybrid system to evaluate microcytic anemia such as IDA, hemoglobinopathy and anemia of chronic disease using HCT, MCV, RDW and obtained 96.5% accuracy for 473 cases of microcytic anemia and anemia of chronic disease. Dogan et al. [70] proposed IDA detection method based on hematology parameters, namely Serum iron and total iron-binding capacity, using decision trees for 96 patients and the results got perfectly matched with the physician’s decisions. Lund et al. [103] presented an algorithm to classify microcytic and macrocytic anemia using image analysis techniques based on MCV and RBC size and obtained 95% accuracy for 4000 cells. Sanap et al. [137] proposed an anemia classification method based on CBC reports using C4.5 decision tree algorithm and SVM using WEKA tool with 514 instances and obtained accuracy of 99.42% and 88.13% respectively. Abdullah et al. [12] presented anemia types prediction method based on CBC reports using data mining techniques using WEKA tool for 41 patients. The experiment showed that the J48 decision tree performed better with 97% precision among Naive Bayes, MLP and SVM algorithms. Jaiswal et al. [94] presented anemia prediction method based on CBC reports using supervised machine learning algorithms. This method used eighteen attributes from 200 samples and obtained maximum accuracy of 96.09%. In this work, it was reported that Naive Bayes outperformed C4.5 and random forest. Khalaf et al. [96] presented machine learning approaches for the classification of SCD dosage levels using 13 attributes from 1168 sample points. They concluded that the random forest classifier performed overall better than RNN and feedforward neural networks. Amendolia et al. [31, 32] presented ANN-based method to detect *α* and β thalassemia using hemochromic parameters. A specialized ANN was used in the method and accuracy of 94% was achieved for 304 cases. Setsirichok et al. [142] proposed a method for classifying thalassemia using Hb and MCV parameters and obtained an average accuracy in the range of 93–99% for 8054 clinical trial samples using C4.5 decision tree, Naive Bayes classifier and MLP. However, they mentioned that Hb parameter is redundant for the study.

Table 4 summarizes the anemia classification methods used in literature.

**Fig. 6:**
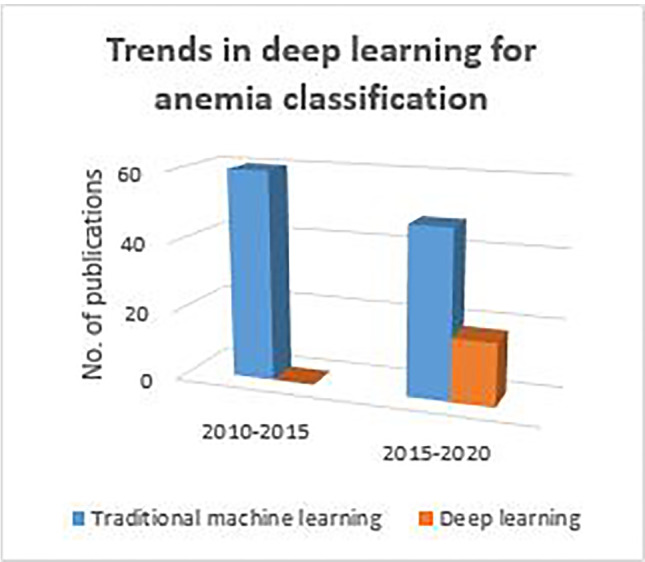
Application of traditional machine learning and deep learning for anemia classification

It is evident from Fig. 6 that, most of the research groups used the traditional machine learning approach for anemia classification. It can also be observed that, there is an increasing tendency towards the usage of deep learning classifier models.

**Table 4:** Anemia classification methods

Methods	No. of images	Accuracy (%)	Remarks	Ref.
GLCM, CNN	256	BPNN—93.2, CNN—92.6 for minor thalassemia case	Sample size is less	[153]
SVM, KNN, MLP	304 records	MLP—92 , SVM—83 sensitivity for thalassemia	Using RBC, Hb, HCT, MCV parameters	[31, 32]
ANN	473 cases	96.5 for IDA, HA, ACD	Using HCT, MCV, RDW	[42]
Active contour, NN, DT	15 groups	82–93 for thalassaemia	False-positive and false negative errors are less than 1% and 2%	[?, 109]
C4.5 DT, Naive Bayes classifier and MLP	8054 samples	99.4 for 18 classes of thalassaemia	Using six Hb attributes and MCV	[142]
Marker-controlled Watershed segmentation, KNN	100	80.6 for SCA and thalassaemia	Developed combined method	[145]
Fuzzy C means clustering, geometrical and statistical features	80	KNN—73.3, SVM—83.3, ELM—87.7 for SCD	Fuzzy C means overcomes the disadvantages of threshold segmentation	[55, 56]
HSI color space, K-means clustering	60	94.6 for thalassemia	Detected *α*, *β* thalassemia, *β*-thalassemia trait	[129]
ANN, GLCM features	100	75–81 for IDA	Classified 4 types of poikilocytes	[152]
CHT, marker-controlled WT, LOG, Fuzzy thresholding	8–20	91.1 for SCD	CHT performed better, need improvement in de-noising method	[13] [21, 22, 88, 114, 115]
CLAHE, MLP and random forest	100 instances	92 for IDA and HA	Persistent results for any luminosity conditions	[35]
Deformable U-Net	266 raw	99.12 for SCD RBC	Method could segment blurred, clustered, heterogeneous shaped RBCs	[163]
Chain codes, Bayes classifier, logistic model trees and rules classifier	24	96.6 for HA	HA is classified based on differential value of chain codes	[52]
Naive Bayes, C4.5 and random forest classifier	200 samples	96.1 for anemia detection	Used 18 attributes from CBC reports	[94]
DL, multi-class SVM	100–250	99.5 for SCD	Proposed three CNN models with different layers and filters	[30]
DL-Alexnet	750 single RBCs	95.9 for SCA	Specificity was low due to less normal cells	[25]
U-net architecture, semantic segmentation	96 unique samples	98 for SCD	Developed smartphone microscope	[85]

**Fig. 7:**
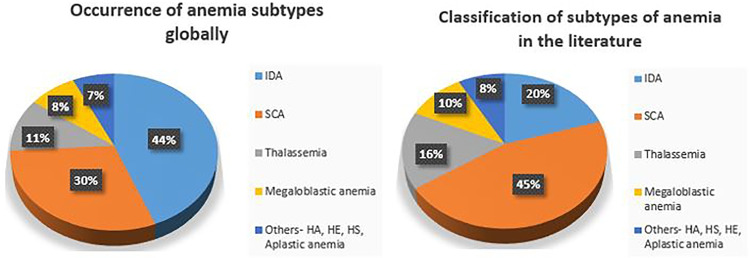
Occurrence and classification of anemia subtypes

Occurrence of anemia worldwide according to WHO [50, 111] and papers on classification of subtypes of anemia are shown in Fig. 7.

Out of many anemia subtypes, the frequency of SCA and IDA detection was high by the majority of the researchers which is depicted in Fig. 7. It can be observed from figure that occurrence of IDA globally is much higher than SCA. However, number of studies seen in the literature is not in the same proportion. There is scope for more study in IDA detection. Also, there is a need for other anemia subtypes detection and diagnosis in peripheral blood smear analysis.

## Database

Many researchers have used proprietary datasets with blood smear images of different stains. A few publicly available databases are used for the performance analysis of the developed algorithm. An overview of these databases are given in Table 5.

**Table 5:** Outline of the publicly available databases

Database	No. of images	Annotations	Studies
BCCD [4]	364 smear images	Available for RBCs, WBCs and platelets	[20]
erythrocytesIDB [6]	196 smear images and 629 Giemsa stained single RBCs	Available for sickle cells of 80 smear images	[29, 81, 134]
ASH image bank [3]	2100 hematologic Leishman stained images	Not available	-
Isfahan MISP [9]	148	Not available	[139]
PHIL [8]	100	Not available	[14]
BBBC [5]	18 biological image sets	Available for RBC’s only	[19]
Telepathology 2012 [10]	Malarial parasite images	Tool available	[156]
LISC [7]	400 Wright-Giemsa stained images	Available for WBC’s from 250 images only	[138]
ALL-IDB [1]	108 smear images and 260 cropped normal and blast single cell images	Available for WBC’s only	[29, 65, 101, 104, 105, 130, 143, 150, 151, 161]

BCCD is a small-scale publicly available dataset [4] that has 364 annotated images for blood cell detection taken originally from *cosmicad* and *akshaylamba* open sources. The erythrocytesIDB [6] contains 196 full field and 629 individual Giemsa stained peripheral blood smear images taken from SCD patients. ASH image bank [2] is a web-based image library that has a collection of hematologic images consists of normal and abnormal blood cells. However, studies have not explored available images for anemia detection. The atlas of hematology [3] provides normal and abnormal Leishman stained blood smear images for the morphological study of cells. Medical Image and Signal Processing (MISP) Research Center and Department of Pathology at Isfahan University of Medical Sciences [9] contributed for the dataset consists of 148 microscopic blood smear images. Public Health Image Library (PHIL) [8] contains a few blood smear images created by the Centers for Disease Control and Prevention (CDC) for reference. The Broad Bioimage Benchmark Collection (BBBC) [5] consists of publicly available image sets such as annotated biological images for the analysis of algorithms. Telepathology 2012 [10] consists of webmicroscope to acquire malarial parasite data along with annotation tool. Leukocyte Images for Segmentation and Classification (LISC) [7] for identification of different WBCs with ground truth for only 250 images. Acute Lymphoblastic Leukemia Image Database (ALL-IDB) [1] is a free, publicly available dataset for the evaluation of segmentation and classification methods. There are two datasets specifically for lymphoblasts detection. ALL-IDB1 consists of 108 blood smear images with labeled lymphocytes taken with 300 to 500 microscope magnifications. ALL-IDB2 is a collection of 260 cropped normal and blast cells that belongs to ALL-IDB1 dataset.

Even though a lot of work has been carried out on PBS images, annotations are not available for the publicly available datasets. A comparison of work would not be fair because ground truth depends on annotation done by individuals.

## Discussion and future scope

**Fig. 8:**
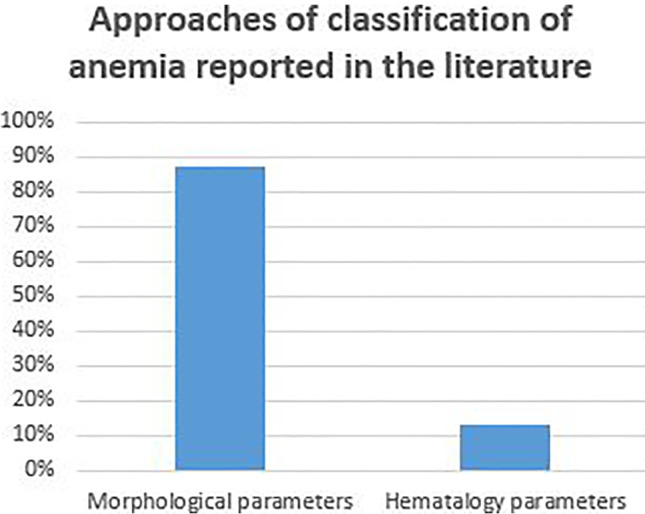
Ways of anemia classification

Diagnosis of anemia is challenging, particularly in inadequate resource settings. Various state-of-the-art methods used in the literature for PBS analysis are mentioned in Section 2 and some of them are listed in Tables 2, 3 and 4. In this paper, we have provided a detailed report of the use of image processing methodologies to automate peripheral blood smear analysis for diagnosing morphology-based RBC disorders. Also, we can notice from Fig. 6 that detection and classification of anemia using deep learning is increasing over traditional machine learning approaches. From Table 5, it is evident that most of the research groups used a proprietary dataset for the analysis of the algorithms. As researchers used a publicly available datasets with different images and annotations, a comparison of the algorithms is not possible to accept the results. It is also observed that the number of images in dataset is in the range from 100 to 1000. As deep learning is becoming popular and many of classification algorithms are supervised learning approaches, it is desirable to have large PBS datasets with annotations. It can be observed from Fig. 7 that the occurrence of IDA is much higher than SCA and studies related to IDA is lesser. Hence, there is a need for more study of IDA and other subtypes of anemia. Figure 8 depicts the ways of classification of anemia. We can observe that around 88% of researchers used morphological parameters and only a few research groups used hematology parameters for the anemia diagnosis.

To detect anemia cases, mostly the shape and size of the abnormal RBCs are considered. The methods used by the research groups did not focus on other abnormalities of blood cells.

It can be noticed that multiple methods have been implemented to detect a few anemia cases like SCA, IDA and thalassemia that mainly depend on the input image taken from different lab setups and conditions. The summary table refers to the papers which are categorized into three divisions depending on the purpose and approaches mentioned in Section 2. All these research groups focused on developing computer-aided automated systems to reduce the task of hematologists in analyzing the peripheral blood smears. A major impediment of automated microscopic evaluation is that they are affected due to imaging and staining variations. An accurate identification of normal and abnormal cases is essential to assist pathologists for further diagnosis. Images are acquired from blood smear stained using a specific stain with different microscopic settings and lab arrangements. However, this poses many challenges for the automation of blood smear images. For example, as mentioned in Section 2 and Table 2, the methods presented by various researchers for RBC segmentation and counting were implemented on specific stained blood smear images under a controlled environment. However, it is observed from the past studies described in the listed papers that results vary due to lack of robustness in the methods. Although an automated decision support system is developed to reduce the burden on hematologists by eliminating manual inspection of blood smears, there is no integrated approach that has been developed to handle both standard and inconsistent microscopic blood smear images acquired from both the manual and automated workflow.

## Conclusion

This review provides a summary of the current developments in computerized PBS analysis using microscopic blood smear images. The paper comprises of introduction to PBS analysis and anemia, an RBC disorder. Different sections have been summarized in this paper on existing automated image processing methods for identification, segmentation, feature extraction and classification of RBCs for further diagnosis of anemia. The literature on approaches of the classification of anemia also has been included in this review. Although, manual microscopic evaluation is a gold standard for PBS analysis, for quick and accurate diagnosis, an automated decision system is essential to overcome the limitations of microscopic analysis. Hence, the observations made during the process of the review are listed below.

- To analyze RBC disorders rigorously, a large publicly available dataset with annotations of various types of blood cells is needed.
- A robust system that can handle staining and imaging variations is desirable in PBS analysis for anemia detection.
- A hybrid method which considers both morphological and clinical features would play an important role to improve the efficiency of classification of anemia subtypes.

An automated system plays a very important role in the PBS analysis. However, it is observed from the review that, there is a need for a large PBS publicly available database with appropriate annotations and robust system to assist clinicians for further diagnosis of disease.
